# Logical fallacies in animal model research

**DOI:** 10.1186/s12993-017-0121-8

**Published:** 2017-02-15

**Authors:** Espen A. Sjoberg

**Affiliations:** Department of Behavioral Sciences, Oslo and Akershus University College of Applied Sciences, St. Olavs Plass, P.O. Box 4, 0130 Oslo, Norway

**Keywords:** Argument from analogy, Confirmation bias, Type 1 error, Animal models, Double-down effect, Validity

## Abstract

**Background:**

Animal models of human behavioural deficits involve conducting experiments on animals with the hope of gaining new knowledge that can be applied to humans. This paper aims to address risks, biases, and fallacies associated with drawing conclusions when conducting experiments on animals, with focus on animal models of mental illness.

**Conclusions:**

Researchers using animal models are susceptible to a fallacy known as false analogy, where inferences based on assumptions of similarities between animals and humans can potentially lead to an incorrect conclusion. There is also a risk of false positive results when evaluating the validity of a putative animal model, particularly if the experiment is not conducted double-blind. It is further argued that animal model experiments are reconstructions of human experiments, and not replications per se, because the animals cannot follow instructions. This leads to an experimental setup that is altered to accommodate the animals, and typically involves a smaller sample size than a human experiment. Researchers on animal models of human behaviour should increase focus on mechanistic validity in order to ensure that the underlying causal mechanisms driving the behaviour are the same, as relying on face validity makes the model susceptible to logical fallacies and a higher risk of Type 1 errors. We discuss measures to reduce bias and risk of making logical fallacies in animal research, and provide a guideline that researchers can follow to increase the rigour of their experiments.

## Logical fallacy

A logical fallacy is a judgment or argument based on poor logical thinking. It is an error in reasoning, which usually means that either the line of reasoning is flawed, or the objects in the premise of the argument are dissimilar to the objects in the conclusion [1]. Scientists are not immune to logical fallacies and are susceptible to making arguments based on unsound reasoning. For instance, a common fallacy is *affirming the consequent*. This involves the following line of reasoning*: if A is true, then X is observed. We observe X, therefore A must be true*. This argument is fallacious because observing X only tells us that there is a possibility that A is true: the rule does not specify that A follows X, even if X always follow A.^1^ Studies that have explicitly investigated this in a scientist sample found that 25–33% of scientists make the fallacy of affirming the consequent and conclude that X → A is a valid argument [2, 3].

Making logical fallacies is a human condition, and there is a large range of fallacies commonly committed [1, 4, 5]. In the present paper, we will focus on a select few that are of particular relevance to animal model research, especially in the context of validity and reliability of conclusions drawn from an experiment.

## Confirmation and falsification

The fallacy of affirming the consequent is connected with a tendency to seek evidence that confirms a hypothesis. Many scientists conduct their experiments under the assumption that their experimental paradigm is a legitimate extension of their hypothesis, and thus their results are used to confirm their beliefs. As an example, imagine a hypothesis that states that patients with bipolar disorder have reduced cognitive processing speed, and we do a reaction time test to measure this. Thus, a fallacious line of reasoning would be: *if bipolar patients have reduced cognitive processing speed, then we will observe slower reaction time on a test. We observe a slower reaction time, and therefore bipolar patients have reduced cognitive processing speed.* This would be affirming the consequent, because the observed outcome is assumed to be the result of the mechanism outlined in the hypothesis, but we cannot with certainty say that this is true. The results certainly suggests this possibility, and it may in fact be true, but the patients may have exhibited slower reaction times for a variety of reasons. If a significant statistical difference between bipolar patients and controls is found, it may be common to conclude that the results support the cognitive processing speed hypothesis, but in reality this analysis only reveals that the null hypothesis can be rejected, not necessarily why it can be rejected [6, 7]. The manipulation of the independent variable gives us a clue as to the cause of the rejection of the null hypothesis, but this does not mean that the alternative hypothesis is confirmed beyond doubt.

Popper [8] claimed that hypotheses could never be confirmed; only falsified. He claimed that we could not conclude with absolute certainty that a statement is true, but it is possible to conclude that it is *not true*. The classic example is the white swan hypothesis: even if we have only observed white swans, we cannot confirm with certainty the statement “all swans are white”, but if we observe a single black swan then we can reject the statement. Looking for confirmation (searching for white swans) includes the risk of drawing the wrong conclusion, which in this case is reached through induction. However, if we seek evidence that could falsify a hypothesis (searching for black swans), then our observations have the potential to reject our hypothesis. Note that rejecting the null hypothesis in statistical analyses is not necessarily synonymous with falsifying an experimental hypothesis. Null-hypothesis testing is a tool, and when we use statistical analyses we are usually analysing a numerical analogy of our experimental hypothesis.

When a hypothesis withstands multiple tests of falsification, Popper called it *corroborated* [9]. We could argue that if a hypothesis is corroborated, then its likelihood of being true increases, because it has survived a gauntlet of criticism by science [10]. However, it is important to note that Popper never made any such suggestion, as this would be inductive reasoning: exactly the problem he was trying to avoid! Even if a hypothesis has supporting evidence and has withstood multiple rounds of falsification, Popper meant that it is not more likely to be true than an alternative hypothesis, and cannot be confirmed with certainty [11]. Instead, he felt that a corroborated theory could not be rejected without good reason, such as a stronger alternative theory [12]. Popper may be correct that we cannot confirm a hypothesis with absolute certainty, but in practice it is acceptable to assume that a hypothesis is likely true if it has withstood multiple rounds of falsification, through multiple independent studies using different manipulations (see “Animal model experiments are reconstructions” section). However, in the quest for truth we must always be aware of the possibility, however slight, that the hypothesis is wrong, even if the current evidence makes this seem unlikely.

### Confirmation bias

Confirmation bias is the tendency to seek information that confirms your hypothesis, rather than seeking information that could falsify it [13]. This can influence the results when the experimenter is informed of the hypothesis being tested, and is particularly problematic if the experiment relies on human observations that has room for error. The experimenters impact on the study is often implicit, and may involve subtly influencing participants or undermining methodological flaws, something also known as *experimenter bias* [14].

The tendency to express confirmation bias in science appears to be moderated by what field of study we belong to. Physicists, biologists, psychologists, and mathematicians appear to be somewhat better at avoiding confirmation bias than historians, sociologists, or engineers, although performance varies greatly from study to study [3, 15–18]. In some cases, the tendency to seek confirming evidence can be a result of the philosophy of science behind a discipline. For instance, Sidman’s [19] book *Tactics of Scientific Research*, considered a landmark textbook on research methods in behavior analysis [20–22], actively encourages researchers to look for similarities between their research and others, which is likely to increase confirmation bias.

Confirmation bias has been shown in animal research as well, but this fallacy is reduced when an experiment is conducted double-blind [23]. Van Wilgenburg and Elgar found that 73% of non-blind studies would report a significant result supporting their hypothesis, while this was only the case in 21% of double-blind studies. An interesting new approach to reduce confirmation bias in animal research is to fully automatize the experiment [24, 25]. This involves setting up the equipment and protocols in advance, so that large portions of an experiment can be run automatically, with minimal interference by the experimenter. Along with double-blinded studies, this is a promising way to reduce confirmation bias in animal experiments.

It is important to note that the confirmation bias phenomenon occurs as an automatic, unintentional process, and is not necessarily a result of deceptive strategies [26]. As humans, we add labels to phenomena and establish certain beliefs about the world, and confirmation bias is a way to cement these beliefs and reinforce our sense of identity.^2^ Scientists may therefore be prone to confirmation bias due to a lack of education on the topic, and not necessarily because they are actively seeking to find corroborating evidence.

## Argument from analogy and animal model research

The issues reported in this paper apply to all of science, and we discuss principles and phenomena that any scientist would hopefully find useful. However, the issues will primarily be discussed in the context of research on animal models, as some of the principles have special applications in this field. In this section, we outline how an animal model is defined, and problems associated with arguing from analogy in animal research.

### Defining an animal model

The term “animal model” is not universally defined in the literature. Here, we define an animal model as *an animal sufficiently similar to a human target group in its physiology or behaviour, based on a natural, bred, or experimentally induced characteristic in the animal, and which purpose is to generate knowledge that may be extrapolated to the human target group.* In this article, we focus on translational animal models in the context of behavioural testing, which usually involve a specific species or strain, or an animal that have undergone a manipulation prior to testing.

An animal model can of course model another non-human animal, but for the most part the aim of it is to study human conditions indirectly through animal research. That research is conducted on animals does not necessarily mean that the animal acts as a model for humans. It is only considered an animal model when its function is to represent a target group or condition in humans, e.g. people with depression, autism, or brain injury. The current paper focuses on animal models of mental illness, but animal models as a whole represent a large variety of conditions, and are particularly common to use in drug trials. See Table 1 for an overview of common animal models of mental illnesses.

**Table 1:** A summary of some available animal models of mental illnesses, where the animals themselves act as the model for the target group

Mental illness	Model	References
Anxiety	Serotonin receptor 1A knockout mice	[114]
	Corticosterone treated mice	[115]
Attention-Deficit/Hyperactivity Disorder	Spontaneously Hypertensive rat	[35]
	Thyroid receptor β1 transgenic mice	[116]
Autism	Valproic Acid rat	[81]
Depression	Corticosterone treated rats and mice	[117]
	Chronic Mild Stress rats and mice	[118]
Obsessive Compulsive Disorder	Quinpirole treated rats	[119]
Post-Traumatic Stress Syndrome	Congenital learned helpless rat	[120]
Schizophrenia	Ventral hippocampus lesioned rats	[121]
	Methylazoxymethanol acetate treated rats	[122]
	Developmental vitamin D deficient rats	[123]

The animals are genetically modified, bred for a specific trait, or manipulated in some physiological fashion (e.g. a lesion or drug injection)

It should also be noted that the term “animal model” refers to an animal model that has at least been validated to some extent, while a model not yet validated is referred to as a “putative animal model”. That a model is “validated” does not mean that the strength of this validation cannot be questioned; it merely means that previous research has given the model credibility in one way or another.

### Arguing from analogy

In research on animal models, scientists sometimes use an approach called the *argument from analogy*. This involves making inferences about a property of one group, based on observations from a second group, because both groups have some other property in common [1]. Analogies can be very useful in our daily lives as well as in science: a mathematical measurement, such as “one meter”, is essentially an analogy where numbers and quantities act as representations of properties in nature. When applying for a job, a person might argue that she would be a good supervisor because she was also a good basketball coach, as the jobs have the property of leadership in common. Concerning animal models, arguing from analogy usually involves making inferences about humans, based on an earlier observation where it was found that the animals and humans have some property in common. Arguing from analogy is essentially a potentially erroneous judgment based on similarities between entities. However, this does not make the argument invalid by default, because the strength of the argument relies on: (1) how relevant the property we infer is to the property that forms the basis of the analogy; (2) to what degree the two groups are similar; (3) and if there is any variety in the observations that form the basis of the argument [1].

Animal models themselves are analogies, as their existence is based on the assumption that they are similar to a target group in some respect. If the two things we are drawing analogies on are similar enough so that we will reasonably expect them to correlate, an argument from analogy can be strong! However, when we draw the conclusion that two things share a characteristic, because we have established that they already share another, different characteristic, then we are at risk of making the *fallacy of false analogy* [27].

### The false analogy

A false analogy is essentially an instance when an argument based on an analogy is incorrect. This can occur when the basis of similarity between objects do not justify the conclusion that the objects are similar in some other respect. For instance, if Jack and Jill are siblings, and Jack has the property of being clumsy, we might infer that Jill is also clumsy. However, we have no information to assert that Jill is clumsy, and the premise for our argument is based solely on the observation that Jack and Jill have genetic properties in common. We are assuming that clumsiness is hereditary, and therefore this is probably a false analogy. Note that knowledge gained later may indicate that—in fact—clumsiness is hereditary, but until we have obtained that knowledge we are operating under assumptions that can lead to false analogies. Even if clumsiness was hereditary, we could still not say with absolute certainty that Jill is clumsy (unless genetics accounted for 100% of the variance). This new knowledge would mean that our analogy is no longer false, as Jill’s clumsiness can probably at least in part be explained by genetics, but we are still arguing from analogy: we cannot know for certain if Jill is clumsy, based solely on observations with Jack.

### The false analogy in animal models

With animal models, the false analogy can occur when one group (e.g. an animal) share some characteristics with another group (e.g. humans), and we assume that the two groups also share other characteristics. For instance, because chimpanzees can follow the gaze of a human, it could be assumed that the non-human primates understand what others perceive, essentially displaying theory of mind [28–30]. However, Povinelli et al. [31] argue that this is a false analogy, because we are drawing conclusions about the inner psychological state of the animal, based on behavioural observations. It may appear that the animal is performing a behaviour that requires complex thinking, while in reality it only reminds us of complex thinking [32], most likely because we are anthropomorphizing the animal’s behaviour [33]—particularly the assumption that the mind of an ape is similar to the mind of a human [30]. A different example would be birds that are able to mimic human speech: the birds are simply repeating sounds, and we are anthropomorphising if we believe the birds actually grasp our concept of language.

Robbins [34] pointed out that homology is not guaranteed between humans and primates, even if both the behavioural paradigm and the experimental result are identical for both species: different processes may have been used by the two species to achieve the same outcome. Since an animal model is based on common properties between the animal and humans, we may assume that new knowledge gained from the animal model is also applicable to humans. In reality, the results are only indicative of evidence in humans.

Arguing from analogy, therefore, involves the risk of applying knowledge gained from the animal over to humans, without knowing with certainty if this application is true. Imagine the following line of reasoning: we find result A in a human experiment, and in an animal model we also find result A, establishing face validity for the animal model. Consequently, we then conduct a different experiment on the animal model, finding result B. If we assume that B also exist in humans, without trying to recreate these results in human experiments, then we are arguing from analogy, potentially drawing a false analogy.

### Illustration: argument from analogy in the SHR model of ADHD

An illustration of argument from analogy comes from the SHR (spontaneously hypertensive rat) model of ADHD (Attention-Deficit/Hyperactivity Disorder) [35, 36]. Compared to controls, usually the Wistar Kyoto rat (WKY), the SHRs exhibit many of the same behavioural deficits observed in ADHD patients, such as impulsive behaviour [37–42], inattention [35, 37], hyperactivity [37, 43], and increased behavioural variability [44–47].

One measure of impulsive behaviour is a test involving delay discounting. In this paradigm, participants are faced with the choice of either a small, immediate reinforcer or a larger, delayed reinforcer. Both ADHD patients [48] and SHRs [41] tend to show a preference for the smaller reinforcer as the delay between response and reinforcer increases for the large reinforcer. Research on delay discounting with ADHD patients suggests that they are *delay averse*, meaning that impulsivity is defined as making choices that actively seek to reduce trial length (or overall delay) rather than immediacy [48–56], but this is usually achieved by choosing a reinforcer with a short delay.

There is no direct evidence to suggest that SHRs operate by the same underlying principles as ADHD patients. Studies on delay discounting using SHRs tend to manipulate the delay period between response and reinforcer delivery, but do not compare the results with alternative explanations. This is because the rats cannot be told the details of the procedure (e.g. if the experiment ends after a specific time or a specific number of responses). Therefore, most authors who have investigated delay discounting usually avoid the term delay aversion [57]. However, some authors make the argument from analogy where they assume that the rats show a similar effect to ADHD children: Bizot et al. [58] concluded that *“…SHR are less prone to wait for a reward than the other two strains, i.e. exhibit a higher impulsivity level…* (p. 220)”, and Pardey, Homewood, Taylor and Cornish [59] concluded that *“…SHRs are more impulsive than the WKY as they are less willing to wait for an expected reinforcer* (p. 170).” Even though the evidence shows that SHRs preference for the large reinforcer drops with increased delay, we cannot conclude with certainty that this occurs because the SHRs do not want to wait. The experimental setup does not tell us anything conclusive about the animal’s motivation, nor its understanding of the environmental conditions. Hayden [60] has argued that the delay discounting task is problematic in measuring impulsivity in animals because it is unlikely that the animals understand the concept of the inter-trial interval. Furthermore, if the SHRs were less willing to wait for a reinforcer, then we may argue that this shows immediacy, and not necessarily delay aversion. In this case, it may instead support the dual pathway model of ADHD, which takes into account both delay aversion and an impulsive drive for immediate reward [56, 61, 62].

Assuming that the rats are delay averse or impulsive is arguing from analogy. The evidence may only suggests that the rats are impulsive, not necessarily why they are impulsive. The results may also not speak to whether the reason for this behaviour is the same in ADHD and SHRs (mechanistic validity—see “Mechanistic validity” section). If we were to manipulate the magnitude of the large reinforcer then we will also find a change in performance [57, 63]. How do we know that the SHRs are sensitive to temporal delays, and not to other changes in the experimental setup, such as the inter-trial interval [60], reinforcer magnitude [63], or the relative long-term value of the reward [64]?

## The validity criteria of animal models

Before any further discussion on logical fallacies in animal models, the validity criteria of these models must be addressed. We must also point out that there are two approaches to animal model research: (1) validating a putative animal model, and (2) conducting research on an already validated model.

When asserting the criteria for validating an putative animal model, the paper by Willner [65] is often cited, claiming that the criteria for a valid animal model rests on its face, construct, and predictive validity. This means that the model must appear to show the same symptoms as the human target group (face validity), that the experiment measures what it claims to measure and can be unambiguously interpreted (construct validity), and that it can make predictions about the human population (predictive validity). However, there is no universally accepted standard for which criteria must be met in order for an animal model to be considered valid, and the criteria employed may vary from study to study [66–70]. Based on this, Belzung and Lemoine [71] attempted to broaden Willner’s criteria into a larger framework, proposing nine validity criteria that assess the validity of animal models for psychiatric disorders. Tricklebank and Garner [72] have argued that, in addition to the three criteria by Willner [65], a good animal model must also be evaluated based on how it controls for third variable influences (internal validity), to what degree results can be generalized (external validity), whether measures expected to relate actually do relate (convergent validity), and whether measures expected to not relate actually do not relate (discriminant validity). These authors argue that no known animal model currently fulfils all of these criteria, but we might not expect them to; what is of utmost importance is that we recognize the limitation of an animal model, including its application. Indeed, it could be argued that a reliable animal model may not need to tick all the validity boxes as long it has predictive validity, because in the end its foremost purpose is to make empirical predictions about its human target group. However, be aware that arguing from analogy reduces the model’s predictive validity, because its predictive capabilities may be limited to the animal studied.

### Mechanistic validity

Behavioural similarities between a putative model and its human target group is not sufficient grounds to validate a model. In other words, face validity is not enough: arguably, *mechanistic validity* is more important. This is a term that normally refers to the underlying cognitive and biological mechanisms of the behavioural deficits being identical in both animals and humans [71], though we can extend the definition to include external variables affecting the behaviour, rather than attributing causality to only internal, cognitive events. Whether the observed behaviour is explained in terms of neurological interactions, cognitive processes, or environmental reinforcement depends on the case in question, but the core of matter is that mechanistic validity refers to the *cause of the observed behavioural deficit or symptom*. If we can identify the cause of the observed behaviour in an animal model, and in addition establish that this is also the cause of the same behaviour in humans, then we have established mechanistic validity. This validity criterion does not speak to what has triggered the onset of a condition (trigger validity), or what made the organism vulnerable to the condition in the first place (ontopathogenic validity), but rather what factors are producing the specific symptoms or behaviour [71]. For instance, falling down the stairs might have caused brain injury (trigger validity), and this injury in turn reduced dopamine transmission in the brain, which lead to impulsive behaviour. When an animal model is also impulsive due to reduced dopamine transmissions, we have established mechanistic validity (even if the trigger was different).

### The validity of models of conditions with limited etiology

Face validity has been argued to be of relatively low importance in an animal model, because it does not speak about why the behaviour occurs [33, 69], i.e. the evidence is only superficial. However, it could be argued that face validity is of higher importance in animal models of ADHD, because the complete etiology underlying the condition is not yet fully known, and therefore an ADHD diagnosis is based entirely on behavioural symptoms [73].

There is limited knowledge of the pathophysiology on many of the mental illnesses in the *Diagnostic and Statistical Manual of Mental Disorders* [74]; depression and bipolar disorder are examples of heterogeneous conditions where animal models have been difficult to establish [75, 76]. When dealing with a heterogeneous mental disorder, it is inherently harder for animal models to mimic the behavioural deficits, particularly a range of different deficits [75, 77–80]. We could argue, therefore, that mechanistic validity in animal models is difficult, if not impossible, to establish from the outset when our knowledge of causality in humans might be limited.

### Models can be holistic or reductionist

Animal models can be approached with different applications in mind: it can aim to act *holistic* or *reductionist*. A holistic approach assumes that the model is a good representation of the target group as a whole, including all or most symptoms and behavioural or neurological characteristics. Alternatively, a reductionist approach uses an animal model to mimic specific aspects of a target group, such as only one symptom. This separation may not be apparent, because animal models are usually addressed as if they are holistic; for instance, the valproic acid (VPA) rat model of autism is typically just labelled as an “animal model of autism” in the title or text [81], but experiments typically investigate specific aspects of autism [82–84]. This does not mean that the model is not holistic, but rather that its predictive validity is limited to the aspects of autism investigated so far. Similarly, the SHR is typically labelled as an “animal model of ADHD” [35], but it has been suggested that the model is best suited for the combined subtype of ADHD [36, 73], while Wistar Kyoto rats from Charles River Laboratories are more suited for the inattentive subtype [85]. The point of this distinction between holistic and reductionist approaches is to underline that animal models have many uses, and falsifying a model in the context of one symptom does not mean the model has become redundant. As long as the model has predictive validity in one area or another, then it can still generate hypotheses and expand our understanding of the target group, even if the model is not a good representation of the target group as a whole. Indeed, an animal model may actually be treated as holistic until it can be empirically suggested that it should in fact be reductionist. However, researchers should take care not to assume that a model is holistic based on just a few observations: this would be arguing from analogy and bears the risk of making applications about humans that are currently not established empirically. The exact applications and limitations of an animal model should always be clearly defined [33, 86].

## Animal model experiments are reconstructions

The terms “replicate” and “reproduce” are often used interchangeably in the literature [87], but with regards to animal models their distinction is particularly important. *Replication* involves repeating an experiment using the same methods as the original experiment, while a *reproduction* involves investigating the same phenomenon using different methods [88]. Replications assure that the effects are stable, but a reproduction is needed to ensure that the effect was not due to methodological issues.

We suggest a third term, *reconstruction*, which has special applications in animal models. A reconstruction involves redesigning an experiment, while maintaining the original hypothesis, in order to accommodate different species. When an animal experiment aims to investigate a phenomenon previously observed on humans, we have to make certain changes for several reasons. First, the animals are a different species than humans, and have a different physiology and life experience. Second, the animals do not follow verbal instructions and must often (but not always) be trained to respond. Third, the experimental setup must often be amended so that a behaviour equivalent to a human behaviour is measured. A fourth observation is that animal studies tend to use smaller sample sizes than human experiments, which makes them more likely to produce large effect sizes when a significant result is found [89].

An animal model experiment actively attempts to reconstruct the conditions of which we observed an effect with humans, but makes alterations so that we can be relatively certain that an equivalent effect is observed in the animals (or vice versa, where a human experiment measures an equivalent effect to what was observed in an animal study). This questions the construct validity of the study: how certain are we that the task accurately reflects the human behaviour we are investigating?

Another problem concerned with reconstruction is the standardization fallacy [90]. This refers to the fact that animal experiments are best replicated if every aspect of the experiment is standardized. However, by increasing experimental control we lose external validity, meaning that the results are less likely to apply to other situations [91]. The difficulty is therefore to find a balance between the two, and finding this balance may depend on the research question we seek to answer [33, 92]. One approach is to initially begin with replications, and if these are successful move on to perform reproductions, and eventually reconstructions. This is essentially what van der Staay, Arndt and Nordquist [92] have previously suggested: successful direct replication is followed by extended replication where modifications are made within the procedure, the animal’s environment (e.g. housing or rearing), or their gender. Should the effect persevere, then we have systematically established a higher degree of generalization without losing internal validity. At the final stage, quasi-replications are conducted using different species, which is similar to our concept of reconstructions, and it is at this stage that the translational value of the findings are evaluated.

## The double-down effect

When we run animal model experiments, we have to use a control group for comparison. When we are evaluating a putative model, we are therefore indirectly evaluating both animal groups for their appropriateness as an animal model for the phenomenon in question, even if we hypothesized beforehand that just one group would be suitable, and this is the *double*-*down effect*. If we were to discover that the control group, rather than the experiment group, shows the predicted characteristic, then it may be tempting to use hindsight bias to rationalize that the result was predicted beforehand, something that should always be avoided! In actuality, this is an occasion that can be used to map the observable characteristics of the animals, which is called *phenotyping*. This may show that the control group has a property that makes them a suitable candidate as a new putative model. Follow-up studies can then formally evaluate whether this putative animal model has validity. This approach is perfectly acceptable, provided that the initial discovery of the control group’s suitability is seen as suggestive and not conclusive, until further study provide more evidence.

When an animal model has already been validated, the double-down effect still applies: we are still indirectly evaluating two animal groups at once, but it is less likely that that the control group will display the animal’s characteristic due to previous validation. Failure to replicate previous findings can be interpreted in many ways; it could be an error in measurement, differences in experimental manipulations, or that the animal model is simply not suitable as a model in this specific paradigm (but still viable in others). Should we observe that controls express a phenomenon that was expected of the experimental group, then we should replicate the study to rule out that the finding occurred by chance or through some methodological error. This may lead us to suggest the control group as a putative model, pending further validation.

### The double-down effect and the file drawer problem

Since the purpose of animal models is to conduct research on non-human animals, with the aim to advance knowledge about humans, then inevitably the animal model and the human condition it mimics must be similar in some respect. If they were not, then the pursuit of the model would be redundant. Therefore, from the outset, there is likely to be publication bias in favour of data that shows support for a putative animal model, because otherwise it has no applications.

The double-down effect of evaluating two animal groups at once makes animal models particularly susceptible to the *file drawer problem*. This is a problem where the literature primarily reflects publications that found significant results, while null results are published less frequently [93, 94]. This aversion to the null creates what Ferguson and Heene called “undead theories”, which are theories that survive rejection indefinitely, because null results that refute them are not published [95]. The origin of this trend is not entirely clear, but it probably came into existence by treating the presence of a phenomenon as more interesting than its absence. Once an effect has been documented, replications may now be published that support the underlying hypothesis.

The file drawer effect is probably related to the *sunk*-*cost effect*: this is a tendency to continue on a project due to prior investment, rather than switching to a more viable alternative [96]. Thus, if we publish null results, it may seem that previous publications with significant findings were wasteful, and we may feel that we are contributing towards dissent rather than towards finding solutions. It may be in the researcher’s interest to find evidence supporting the theory in order to justify their invested time, thus becoming victim of confirmation bias.

Furthermore, if null results are found, they might be treated with more skepticism than a significant result. This is, of course, a fallacy in itself as both results should be treated the same: why would a null result be subjected to more scrutiny than a significant result? When the CERN facility recorded particles travelling faster than the speed of light, the observation appeared to falsify the theory of relativity [97]. This result was met with skepticism [98], and it was assumed that it was due to a measurement error (which in the end it turned out to be). Nevertheless, if the result had supported relativity, would the degree of skepticism have been the same?

In the context of animal studies, the double-down effect makes it more likely that a significant result is found when comparing two animal groups. Either group may be a suitable candidate for a putative animal model, even if only one group was predicted to be suitable beforehand. If any result other than a null result will show support for an animal model (or a putative model), then multiple viable models will be present in the literature, all of which will be hard to falsify (as falsifying one model may support another). Indeed, this is currently the case for animal models, where there are multiple available models for the same human conditions [80, 99–103]. The file drawer problem is a serious issue in science [104], and the trend may often be invisible to the naked eye, but methods such as meta-analyses have multiple tools to help detect publication bias in the literature [105].

## Measures to improve animal model research

The main purpose of this paper was to address several risks and fallacies that may occur in animal model research, in order to encourage a rigorous scientific pursuit in this field. We do not intend to discourage researchers from using animal models, but rather hope to increase awareness of potential risks and fallacies involved. In order to make the issues addressed in the paper more overviewable, we have created a list for researchers to confer when designing animal experiment and interpreting their data.

1. *Be aware of your own limitations*. Some of the fallacies and risks addressed in this paper may be unavoidable for a variety of reasons. Nevertheless, the first step towards improving one’s research is to be aware of the existence of these risks. When writing the discussion section of a report, it may be necessary to point out possible limitations. Even if they are not explicitly stated, it is still healthy for any scientist to be aware of them.^3^
2. *Establish predictive and mechanistic validity.* If you are attempting to validate a putative animal model, ensure that the experiment is as similar as possible to experiments done on humans. If this is not possible, explain why in the write-up. If the experiment is novel, and the animal model is already validated through previous research, then this principle does not necessarily apply, because the purpose is to uncover new knowledge that may be translated to humans. In such instances, a new hypothesis gains validity in a follow-up experiment on humans.
  Remember that there are several criteria available for validating an animal model, but there is no universal agreement on which set of criteria should be followed. However, the two most important criteria are arguably predictive validity and mechanistic validity, because face validity is prone to logical fallacies. Establishing mechanistic validity ensures that the mechanisms causing the observed behaviour are the same in the model and humans, while establishing predictive validity means that knowledge gained from the model is more likely to apply to humans.
3. *Define an a priori hypothesis and plan the statistical analysis beforehand*. It is crucial to have an a priori hypothesis prior to conducting the experiment, otherwise one might be accused of data dredging and reasoning after-the-fact that the results were expected [107, 108]. When validating a putative animal model, this drastically reduces the double-down effect. If the data do not show the predicted pattern then it is perfectly acceptable to suggest a new hypothesis and/or a putative animal model for further research.
  When designing the experiment, keep in mind which statistical analysis would be appropriate for analysing the data. If the statistical method is chosen post hoc, then it may not correspond to the chosen design, and one might be accused of data dredging, which involves choosing a statistical procedure that is more likely to produce significant results [107]. Also, keep in mind which post hoc tests are planned, and that the correct one is chosen to reduce familywise error when there are multiple comparisons to be made. It is highly recommended that effect sizes are reported for every statistical test: this will give insight into the strength of the observed phenomenon, and also allow a more detailed comparison between studies [109].
4. *Do a power analysis*. For logistical, practical, or economic reasons, animal model research may be forced to use sample sizes smaller than what is ideal. Nevertheless, one should conduct a power analysis to ascertain how many animals should be tested before the experiment starts. When doing multiple comparisons, it may be difficult to establish the sample size because the power analysis may only grant the sample size of an omnibus analysis (the analysis of the whole, not its individual parts), and not what is required to reach significance with post hoc tests [110]. If all the post hoc analyses are of equal interest, choose the sample size required to achieve power of 0.8 in all comparisons. Alternatively, use a comparison-of-most-interest approach where the sample size is determined by the power analysis of the post hoc comparison that is of highest interest [110]. If a power analysis is not conducted, or not adhered to, it may be prudent to use a sample size similar to previously conducted experiments in the literature, and then do a post hoc power analysis to determine the power of your study. Once the experiment is completed and the data analysed, one must never increase the sample size, because this will increase your chances of finding a significant result (confirmation bias) [109, 111, 112].
5. *Double-blind the experiment*. By doing the experiment double-blind, we severely reduce the risk of confirmation bias. This means that the experimenter is blind to the a priori hypothesis of the study, as well as what group each animal belongs to. However, in some cases it may be difficult or impossible to do this. For instance, if the experimental group has a phenotype that distinguishes them from controls (e.g. white vs. brown rats), then it is difficult to blind the experimenter. For logistical and monetary reasons it may also be impractical to have a qualified experimenter who is blind to the relevant literature of the study. Also, avoid analysing data prior to the experiment’s completion, because if the data are not in line with your predictions then one might implicitly influence the experiment to get the data needed (experimenter bias [14]). Be aware that it is nevertheless perfectly acceptable to inspect the data on occasion without statistically analysing it, just to ensure that the equipment is working as it is supposed to (or state in advance at what point it is acceptable to check the data, in case there are circumstances where you may want to terminate the experiment early).
6. *Avoid anthropomorphizing*. While it is inevitable to describe our results in the context of human understanding and language, we must be careful not to attribute the animals with human-like qualities. Avoid making inferences about the animal’s thoughts, feelings, inner motivation, or understanding of the situation. We can report what the animals did, and what this means in the context of our hypothesis, but take care not to make assumptions of the inner workings of the animal.
7. *Avoid arguing from analogy*. No matter how validated an animal model is, we cannot be certain that a newly observed effect also applies to humans. If research on an animal model yields new information that could give insight into the human target group, ensure to mention that the data is suggestive, not conclusive, pending further validation. Remember that the strength of an animal model is to generate new knowledge and hypotheses relevant to the target group, including the assessment of potentially useful treatments, but that these new possibilities are only hypothetical once they are discovered.
8. *Attempt to publish, despite a null result*. If you predicted a specific result based on trends in the literature, but failed to find this result, do not be discouraged from publishing the data (especially if you failed to replicate a result in a series of experiments). This is particularly important if the experiment had a low sample size, as null results from such studies are probably the least likely to be published, thus fuelling the file drawer problem. By making the data available via either an article (for instance through *Journal of Articles in Support of the Null Hypothesis*) or a dataset online, then you are actively contributing to reduce the file drawer problem.
9. *Replicate, reproduce, and reconstruct.* Replicating an experiment in order to establish interval validity and reliability of an animal model is essential. When replicating experiments multiple times, we reduce the risk that the original finding was a chance result. If previous replications have succeeded, then attempt to include a new hypothesis, experimental manipulation, or follow-up experiment during the study to expand our knowledge of the research question. This process establishes both internal and external validity. Finally, reconstruct the experiment on humans, so that the findings may be attributed across species.

## A note on neurological similarities

The principles discussed in this paper have been addressed in a behavioural context, but it should be noted that they also apply to neurological evidence for animal models, though increasing the validity in this case can operate somewhat differently.

When we find neurological elements that are the same in both the animal model and the human target group (that do not exist in controls), we should be careful to draw any conclusions based on this. Just like behavioural evidence, the links are suggestive and not necessarily conclusive. It is risky to assume that the physiological properties shared between humans and animals operate the same way. In drug research, over 90% of drugs that show effectiveness on animal models fail to work on humans, a problem called *attrition* [113]. In the context of animal models of mental illness, Belzung and Lemoine [71] proposed the concept *biomarker validity*, which means that the function of a neurological mechanism is the same in the animal model and humans, even if the biomarker responsible for this function may be different across the species. In other words, the two species may have different biological markers, but as long as they operate the same way, and in turn produce the same symptoms, then this adds validity to the model.

Of course, in reality things are not this simple. Neurological evidence is usually not based on the presence of a single component, but rather multiple elements such as rate of neurotransmitter release, reuptake, polymorphism, neural pathways, drug effectiveness, or a combination of factors. The core message is that we must be aware that finding similar neurological elements in both animals and humans does not mean that they operate the same way. If we make this assumption, we are arguing from analogy.

It should be noted that confirmation bias could also be a problematic issue in neuroscientific research. Garner [113] illustrates this with a car example: if we believe that the gas pedal of a car is the cause of car accidents, then removing the gas pedal from a car will drastically reduce the accident rate of that car, confirming that indeed the gas pedal was the cause of car accidents. In neuroscience, we may knock out a gene or selectively breed strains to add or remove a genetic component. When the hypothesized behaviour is shown (or not shown), we might conclude that we have confirmed our hypothesis. The conclusion could be wrong because it is based on correlation, and thus future replications of this result is likely to make the same logical error [113].

## Closing remarks

In this paper, it has been discussed how animal models can be susceptible to logical fallacies, bias, and a risk of getting results that could give a false sense of support for a putative animal model. Researchers should remember that behavioural results found in an animal model of a human condition does not guarantee that this knowledge is applicable to humans. Replicating, reproducing and reconstructing results over numerous studies will drastically reduce the probability that the results are similar by chance alone, although this does not necessarily shed light on why the behaviour occurs. Researchers should therefore be encouraged to investigate mechanistic validity, meaning what underlying processes are causing the behaviour. By simply looking at face validity, we have an increased risk of making errors through comparisons.

Animal models can be very useful for investigating the mechanisms behind a human condition. This new knowledge can help improve our understanding and treatment of this condition, but the researcher must not assume that the observed animal behaviour also applies to humans. Ultimately, animal models only provide solid evidence for the animal used, and indicative evidence of human behaviour. However, this is also the strength of animal models: indicative evidence may open the door to new ideas about human behaviour that were not previously considered. Through reconstructions, it can be established whether or not the phenomenon exists in humans, and if the model has mechanistic validity and predictive validity then this certainly increases the application of the model, as well as its value for the progress of human health.

## Abbreviations

ADHD: Attention-Deficit/Hyperactivity Disorder
CERN: European Organization for Nuclear Research
DSM: Diagnostic and Statistical Manual of Mental Disorders
SHR: spontaneously hypertensive rat
VPA: valproic acid rat
WKY: Wistar Kyoto rat

## Footnotes

1 If you struggle to follow this line of reasoning, a concrete example makes it easier: *If it is wine, then the drink has water in it. Water is in the drink. Therefore, it must be wine*. Nowhere does the rule specify that only wine contains water as an ingredient, so simply making this observation does not allow us to conclude that it is wine.

2 Thanks to Rachael Wilner for pointing out this argument.

3 The author of this manuscript once held a conference talk where he suggested the possibility that one of his own research results may have been influenced by confirmation bias [106]. Never assume that only others are prone to bias—even authors of logical fallacy papers may commit fallacies!.
